# Comparative Efficiencies of TiO_2_ Photocatalysts on β-Blocker Metoprolol Degradation by Solar Heterogeneous Photocatalysis

**DOI:** 10.3390/nano15181445

**Published:** 2025-09-19

**Authors:** Irma C. Torrecillas-Rodríguez, Francisco Rodríguez-González, Daniel Tapia-Maruri, Héctor J. Dorantes-Rosales, José L. Molina-González, Cynthia M. Núñez-Núñez, José B. Proal-Nájera

**Affiliations:** 1 CIIDIR- Durango, Instituto Politécnico Nacional, Durango C.P. 34220, Durango, Mexico; 2 Centro de Desarrollo de Productos Bióticos, Instituto Politécnico Nacional, Yautepec C.P. 62731, Morelos, Mexico; 3 Escuela Superior de Ingeniería Química e Industrias Extractivas, Instituto Politécnico Nacional, Ciudad de Mexico C.P. 07300, Mexico; 4 Centro de Investigación y Desarrollo Tecnológico del Agua, Universidad de Salamanca, 37080 Salamanca, Spain; 5 Ingeniería en Tecnología Ambiental, Universidad Politécnica de Durango, Durango C.P. 34300, Durango, Mexico

**Keywords:** anatase, rutile, flat-plate reactor, solar radiation, band gap, wastewater, advanced oxidation process, nanostructures

## Abstract

The degradation of metoprolol (MET) has become a topic of interest due to its persistence in the environment. TiO_2_ is a catalyst commonly used for the degradation of emergent pollutants through photocatalysis due to its physicochemical properties, and it has been pointed out that its crystallite structure and size affect the photocatalytic efficiency. In this study, three brands of TiO_2_ (Evonik P25, Fermont and Sigma Aldrich) were characterized to evaluate their crystallographic and morphological properties. Then, their photocatalytic capacity was tested in solar heterogeneous photocatalysis experiments when degrading MET under various experimental conditions. The TiO_2_ catalysts tested yielded different results when degrading MET in photocatalytic experiments, indicating that presence of a rutile phase in the catalyst and the crystal size are important factors for the success of this semiconductor. Results from solar heterogeneous photocatalysis for MET degradation indicate efficiencies as P25 > Sigma-Aldrich > Fermont, but demonstrate that, even lower-priced TiO_2_ catalysts yield good results for contaminant degradation (90% MET degradation for P25 against 63% when using Sigma Aldrich TiO_2_). This study highlights the potential of solar photocatalysis with lower-priced TiO_2_ catalysts as a viable and sustainable solution for the decontamination of pharmaceutical wastewater in large scale photocatalytic applications.

## 1. Introduction

Metoprolol (MET) is a drug belonging to the β-blocker family; these drugs block the beta (β) receptors that are present in the myocardial tissue of the heart (Figure S1). This drug is a cardio-selective beta 1-adrenergic blocker, which means that it will only act on the β1receptor, thus reducing inotropism (force of contraction of the heart) and chronotropism (heart rate). MET is administered orally and is found in compressed tablets at concentrations ranging from 50 to 100 mg [1]. It is used mainly in adults as a treatment for high blood pressure, although it is also used to treat angina pectoris, cardiac arrhythmias, functional heart problems with palpitations, migraine prophylaxis, and other related conditions. The drug is released within approximately 20 h after ingestion, and its effect lasts up to 24 h. Complete absorption occurs after oral administration, and the substance is absorbed throughout the entire gastrointestinal tract, including the colon. The bioavailability of MET is 30–40%. Approximately 5% of MET is excreted unchanged by the kidney, the remaining dose is excreted in the form of metabolites [2].

Due to the incorrect disposal of waste from the pharmaceutical, veterinarian and farm industries, excretion from humans and animals after drug administration, and the inability of current wastewater treatment plants (WWTPs) to degrade organic pollutants, beta-blocker drugs such as MET have been detected in wastewater treatment plant (WWTP) effluents at concentrations ranging from 0.017 µg L^−1^ to 0.73 mg L^−1^ and even in surface water bodies (1.5 µg L^−1^ to 0.45 mg L^−1^) [3,4,5,6] and groundwater. Pharmaceuticals have been recently classified as emerging contaminants, since their negative impact on the environment was previously unknown. Nevertheless, as compounds with a different origin and chemical nature that tend to go unnoticed, their concentration in the environment is not considered significant [7]. However, this does not exempt them from causing a major ecological impact. A study carried out by Dzialowski et al. [8] reported that upon exposure to 1 to 12 mg L^−1^ of MET, the heartbeat and metabolic rate of *D. magna* decreased; exposure to 50 mg L^−1^ even caused the death of this organism. Also, it has been pointed out by some authors that beta-blockers can alter the levels of testosterone in some organisms (*O. latipes, P. antipodarum*); therefore, these drugs are considered endocrine disruptors [9,10,11].

The inefficiency of current WWTP methods in degrading pharmaceutical drugs is due to the fact that these methods were designed to remove other type of compounds (organic matter, suspended solids, pathogens, etc.). Therefore, current WWTP treatments lack techniques capable of degrading other types of pollutants (e.g., organic compounds) and monitoring programs able to detect concentrations below the ng L^−1^ range [12].

In the last two decades, various Advanced Oxidation Processes (AOPs) have been used to degrade MET in aqueous solution. Abramovic et al. [13] achieved complete mineralization of the drug in 360 min using ultraviolet (UV) photocatalysis, while Moctezuma et al. [14] obtained complete degradation of the drug in 90 min also using UV photocatalysis. On the other hand, Wilde et al. [15] obtained a 63.4% degradation of MET by Ozonation, and later, Kovács et al. [16] conducted a study regarding the degradation of three β-blocker compounds (MET, Atenolol, and Propranolol) using an AOP based on the Sulfate radical where they established that these methods are undoubtedly the most effective for the complete degradation of these drugs.

Photolysis and photocatalysis are two AOPs based on the use of UV radiation (<400 nm) to eliminate contaminants contained in aqueous solution; they absorb the radiation, consequently causing reduction–oxidation reactions, which are essential for the complete degradation of the contaminant. This process is also named mineralization (to obtain CO_2_ and water as a final product), with the only difference being the use of a catalyst during photocatalysis, which will also react with UV radiation to accelerate and improve the pollutant degradation process [17].

TiO_2_ is the catalyst most commonly used in photocatalysis for the degradation of emergent pollutants due to its physicochemical properties (it activates with UV light, can work within a wide pH range, and is resistant to chemical decomposition and photo-corrosion); moreover, it is non-toxic and cheap. TiO_2_ presents three different crystallite structures: anatase, rutile, and brookite. Depending on its synthesis method, a TiO_2_ catalyst can have one, two, or the three structures at different percentages. Anatase and rutile are the structures most commonly present in most TiO_2_ catalysts. It is important to mention that the crystallite structure and size of TiO_2_ affects the photocatalytic degradation of pollutants [18,19,20,21].

The objective of this paper is to assess the degradation of MET in aqueous solution via solar photolysis and solar photocatalysis using three different TiO_2_ catalysts (Evonik P25, Sigma-Aldrich ( St. Louis, MO, USA), and Fermont), comparing their MET removal efficiency based on their synthesis method, anatase–rutile composition (%), and particle size. To our knowledge, no similar studies exist on the photocatalytic degradation of metoprolol with such a combination of characteristics: heterogenous processes, solar radiation, and three brands of TiO_2_ catalyst with different crystallographic and morphological properties. In addition, experimental parameters such as different pH levels and different H_2_O_2_ doses were evaluated.

## 2. Materials and Methods

### 2.1. Chemical Reagents

Metoprolol tartrate (CAS 56392-17-7) was used to obtain the absorbance spectra through UPLC analysis using a mixture of Trifluoroacetic Acid (C_2_HF_3_O_2_) (CAS 76-05-1) and Acetonitrile (C_2_H_3_N) (CAS 75-05-8) as the mobile phase. NaOH (CAS 1310-73-2) and HNO_3_ (CAS 7697-37-2) were used for pH adjustment. These five reagents were purchased from Sigma Aldrich (St. Louis, MO, USA). H_2_O_2_ 30% Solution (CAS 7722-84-1) was purchased from Productos Químicos Monterrey, S.A. de C.V. (Monterrey, Mexico).

For photolysis and photocatalysis experiments, commercial MET tartrate was purchased from SANDOZ (Mexico City, Mexico) as 100 mg tablets.

Three different commercial TiO_2_ brands were tested: TiO_2_ Fermont (CAS 13463-67-6, 99.5% purity), obtained from a national distributor (Productos Químicos Monterrey, S.A. de C.V. FERMONT, Monterrey, Mexico); TiO_2_ Evonik P25 (CAS 13463-67-7, 99.8% purity, now on referred only as P25), from Evonik Industries (Evonik Industries AG, Essen, Germany); and TiO_2_ from Sigma-Aldrich (CAS 1317-70-0, 99.8% purity).

### 2.2. Characterization of Catalysts

Different commercial formulations of TiO_2_ were evaluated, as their properties can vary considerably, despite being nominally the same material. Factors such as crystal phase composition, surface area, and particle size have a direct impact on photocatalytic activity. By comparing catalysts, it was possible to identify which one performed best under the same experimental conditions.

The catalysts underwent a characterization process. The first was X-ray diffraction (XRD) to identify the phases present in the catalyst: anatase or rutile. The X-ray diffraction pattern was obtained using a Bruker D8 advance diffractometer at a wavelength Cu-kα= 1.5406 Å. It was measured in the angular range 15° to 110°, with a step of 0.05° and a time per point of 2 s. Bragg–Brentano geometry was also used with a Ge (111) Johansson monochromator in the primary beam and a LynxEye detector in the secondary beam [22].

UV-Vis/NIR spectrophotometry with an integrating sphere was used to obtain an absorbance spectrum at room temperature, as well as to calculate the energy (eV) band gap using the Kubelka–Munk function. A Perkin Elmer model Lambda 950 UV/Vis/NIR spectrophotometer was used to perform this part of the catalyst characterization.

The morphology of the catalysts was investigated through scanning electron microscopy (SEM) using a JSM-6701F (JEOL, Akishima, Japan) operated at 5 kV and transmission electron microscopy (TEM) using a JEOL 2000FX operated at 200 kV.

### 2.3. Photolysis and Photocatalysis Degradation Experiments

UV-Vis absorption spectra from reagent-grade MET tartrate were first obtained to identify its overlap with visible solar radiation. An isocratic analysis was carried out using Acquity Waters UPLC with a Diode Array Detector (DAD) using two solutions at concentrations of 50 mg L^−1^ and 10 mg L^−1^ of MET tartrate diluted in deionized water. As mobile phases, 0.05% Trifluoroacetic Acid and Acetonitrile were used. The elution gradient used in UPLC analysis is shown in Table S1.

**Figure 1:**
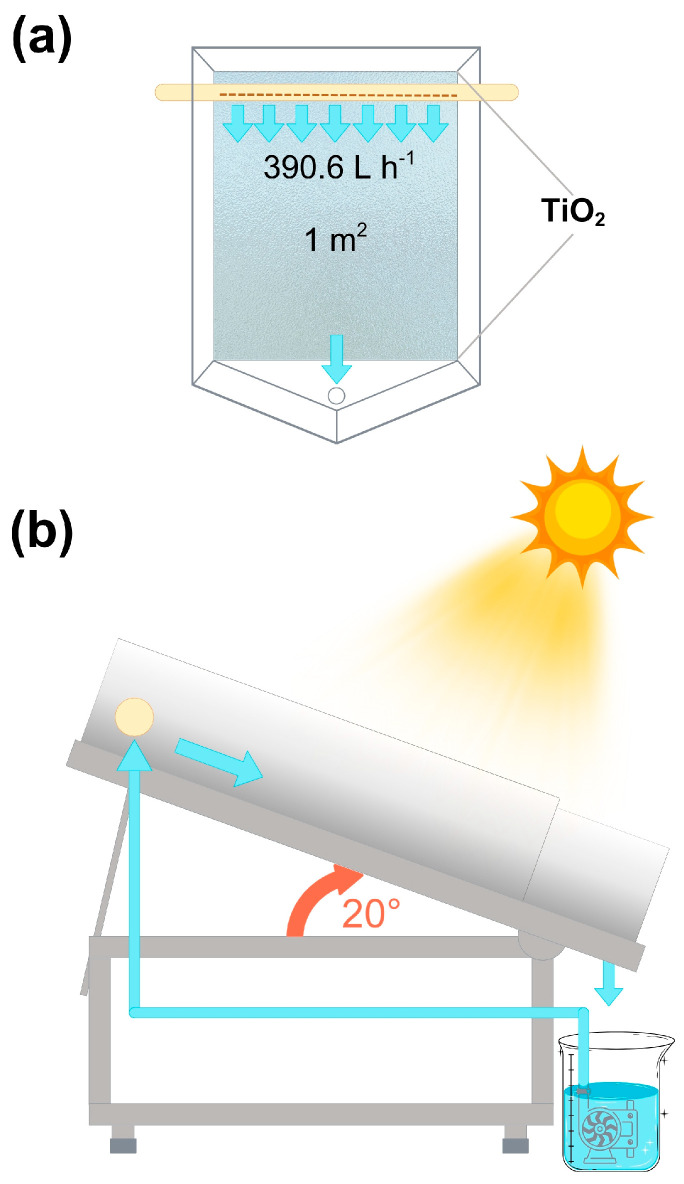
FPR used in the photocatalytic degradation of MET: (**a**) upper view and **(b**) side view.

Heterogeneous photocatalytic and photolytic experiments were carried out in a Flat-Plate Reactor (FPR) with a contact area of 1 m^2^ (Figure 1). The reactor consists of a metal frame (1.30 m length, 0.90 m width, and 1.20 m height) that supports an acrylic bed where the frosted glass plate, to which the catalyst is attached, is inserted. In addition, it has a PVC pipe at the top with holes spaced 0.5 cm apart for the water to flow during the experiment. The contaminated water runs over the glass impregnated with the catalyst, where it receives direct sunlight, and ends up in an opening at the bottom of the reactor. It then accumulates in a container, from where it is recirculated to the reactor by means of a 5 W Bio Pro pump, with a water flux fixed at 390.6 L h^−1^.

The reactor has a 20° inclination, based on the latitude (24.03°) of the city of Durango, Mexico, where the experiments were conducted, and was placed facing the sun. The experimentation was carried out between 12 and 16 h, as radiation is higher during that period (Figure S2), on sunny days with wind speeds of less than 15 km h^−1^.

Radiation data was collected using a WE300 Solar Radiation Sensor pyranometer (GlobalWater, Yellow Springs, OH, USA), which belongs to the meteorological station Secretaría de Recursos Naturales y Medio Ambiente of Durango city. As only around 5% of solar radiation is UV light [23], it was important to perform experiments at the times of the day with the highest solar radiation to increase the number of photons with enough energy to excite catalyst electrons. A series of control experiments to test the effect of each of the parameters was also conducted in the three pH magnitudes, with and without H_2_O_2_ addition, and in the dark.

#### 2.3.1. Effect of Different TiO_2_ Structures on COD Removal

Experiments were performed using TiO_2_ formulations from Fermont, P25, and Sigma Aldrich to compare their behavior during the degradation of pollutants in aqueous solution based on their different crystal structure (anatase–rutile) compositions, particle sizes, and band gaps (eV). Also, a round of experiments was carried out in the absence of catalysts (photolysis experiments). In experiments where TiO_2_ was added, catalyst impregnation on the frosted glass plate was performed by simple deposition. For this, 2 g of TiO_2_ was diluted in 50 mL of distilled water. The solution was then spread evenly over the glass surface, allowing the water to evaporate at room temperature and forming a layer of adsorbed catalyst on the glass surface. The glass plate with the catalyst was washed at the end of each experiment and freshly prepared with the required catalyst before the next one. In photolysis experiments, the frosted glass that was used was clean and dry.

Samples were taken at times of 0, 5, 10, 15, 20, 30, 45, 60, and 80 min in each experiment to determine COD (response variable) and calculate kinetic and statistical data. COD was measured using the HACH Method 8000. The best experiments were also analyzed for TOC using a Teledyne Tekmar TOC Torch analyzer (Teledyne Technologies Incorporated, Thousand Oaks, CA, USA) to check for pollutant mineralization.

#### 2.3.2. Effect of H_2_O_2_ Addition

As electron-hole recombination represents a problem in photocatalysis experiments, the addition of an oxidative reactant to avoid such recombination was tested; thus, experiments were performed in the absence of an oxidant or with the addition of 4 mM H_2_O_2_ per liter of solution (4 mM). For this purpose, when required, the oxidant was added prior to the start of water recirculation. Once added, recirculation started and the initial sample required to quantify the decrease in COD was taken.

The general mechanism for reactive radical species formation by photocatalysis starts with superoxide generation through reduction reactions that take place on the conduction band:

O_2_ + e^−^ → O_2_^−^ (1)

O_2_ + 2e^−^ → O_2_^2−^ (2)

Reactions of oxidation take place in the valence band, and, in the presence of water, HO· radicals are generated:

H_2_O + h^+^ → HO· + H^+^ (3)

HO^−^ + h^+^ → HO∙ (4)

The reaction between some intermediate products can also produce HO

2O_2_^−^+ 2H^+^ → H_2_O_2_ + O_2_ (5)

H_2_O_2_ + e^−^ → HO· + HO^-^ (6)

The HO· formed reacts with organic compounds R present in the sample, something that is known as a mineralization reaction:

HO∙ + R → CO_2_ + H_2_O (7)

#### 2.3.3. Effect of Initial pH

The final factor tested was the initial pH of the solution, as it is well known that the pH of the solution affects efficiency. The TiO_2_ surface charge depends on the point of zero charge (PZC), which is close to neutral pH. Therefore, three pH values were selected: 4.2 (below the PZC, TiO_2_ positively charged), 6.4 (around the PZC, nearly neutral), and 9.2 (above the PZC, TiO_2_ negatively charged). The initial pH was adjusted using NaOH or HNO_3_ solutions. A COD sample was taken after pH adjustment and, when applicable, after H_2_O_2_ addition. To adjust initial pH, NaOH and HNO_3_ solutions were used. The initial COD sample was taken after pH adjustment and, when used, after H_2_O_2_ addition.

### 2.4. Kinetic Parameters Calculation

Data analysis was performed for the best experimental results obtained using a specific catalyst and pH value. Reaction constants (k) were estimated following a first-order reaction, according to Equation (8), using initial reaction times as the data:

The first-order reaction is as follows:

k = (1/t) ln (COD_0_/COD_t_) (8)

where t represents time of reaction, COD_0_ is the initial COD concentration, and COD_t_, is the COD measured at time t.

The half-life time (t_1/2_) was calculated according to Equation (9):

t_1/2_ = (ln 2)/k (9)

### 2.5. Statistical Analysis

To evaluate the effects of factors and covariables involved in the photocatalytic degradation of MET (measured through COD removal), an ANCOVA (Equation (10)) was performed using the statistical software SAS Studio (SAS Studio Basic version 9), using α = 0.05 to determine which parameters were significant in the MET degradation.

COD_ijklm_ = μ + H_2_O_2 i_ + CAT_j_ + pH_k_ + a × T_1_ + b × T_2_ + c × r + DQO_0_ + t_l_ + E_ijklm_ (10)

where COD represents the response variable and the COD measured at sampling time (t) in an experiment performed under an initial (T_1_) and final temperature (T_2_). Equation (10) also considers the catalyst used (CAT), initial pH magnitude (pH), H_2_O_2_ addition, initial sample COD (COD_0_), model mean (μ), and general error (E).

A mean difference analysis was performed through an LSD test to demonstrate the different effects of the catalyst used in photocatalytic experiments. Also, response surface graphs were created in order to better understand the influence of the tested factors.

## 3. Results and Discussion

### 3.1. Catalyst Characterization

The choice of these catalysts allowed us to robustly demonstrate how the combination of crystal structure, morphology, and particle size directly influences the degradation efficiency. The EDS analysis confirmed that elemental impurities (Si, P) are present in minimal percentages, which validates that the performance is primarily determined by the crystallographic and morphological properties of the TiO_2_. Furthermore, the selection of the three TiO_2_ catalysts—Evonik P25, Sigma-Aldrich, and Fermont—was intentional to show that the differences in photocatalytic efficiency are directly attributable to their intrinsic properties, such as crystal structure, particle size, and morphology.

The P25 catalyst was chosen as the benchmark due to its well-known superior efficiency. XRD analysis confirmed a mixed composition of 85.27% anatase and 14.73% rutile. TEM analysis showed that P25 has an average particle size of 11.68 nm, which correlates with a small crystal size (20.97 nm for anatase and 33.96 nm for rutile). This reduced particle size provides a larger specific surface area, which translates into more active sites for contaminant adsorption and redox reactions.

TiO_2_ from Sigma-Aldrich was selected to serve as an intermediate in the comparison. XRD analysis showed that it is predominantly anatase (96.81%), with a minor percentage of rutile (3.19%). SEM micrographs revealed that its morphology is an intermediate point between the fine dispersion of P25 and the larger aggregates of Fermont. Its crystal size is 58.6 nm, which is significantly larger than that of P25. This larger size and lower rutile content explain its intermediate performance in metoprolol degradation, achieving 63% removal, whereas P25 reached 90%.

The Fermont TiO_2_, a low-cost and locally available catalyst, is 100% anatase and has a crystal size of 80.71 nm (the largest of the three). SEM micrographs showed that the particles form dense, globular aggregates, which reduces the available active surface area. The absence of the rutile phase and the smaller available active surface area limited its efficiency.

The crystallographic properties of the three commercial TiO_2_ catalysts tested are presented in Table 1.

XRD patterns and band gap calculations for the Fermont and P25 TiO_2_ catalysts have been published elsewhere by this research group [25].

**Figure 2:**
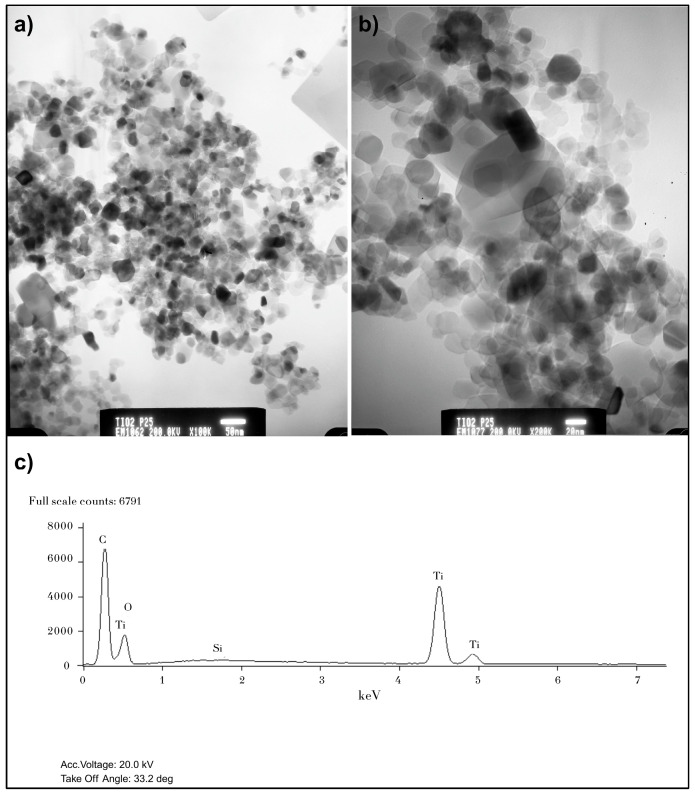
Characterization of P25 catalyst: (**a**) TEM image with a 50 nm scale, (**b**) TEM image with a 20 nm scale, and (**c**) EDS analysis results.

Figure 2 presents transmission electron micrographs and EDS results for P25. The P25 TEM micrographs (Figure 2a,b) show nanoparticles with a predominantly spherical or slightly irregular morphology. A size distribution and a tendency towards agglomeration (forming larger aggregates) are observed. The 50 nm and 20 nm scales allow for the visualization of individual particles and their interactions. Particle size, as observed by TEM/SEM, can be a single crystal or an aggregate of several crystallites. Furthermore, TEM images suggest that primary particles could be in the 20–40 nm range, which is consistent with crystallite sizes. The average particle size obtained was 11.68 nm ± 1.92 nm; this result suggests the presence of smaller primary particles compared to the average crystal sizes reported in Table 1 for the P25 catalyst.

Particles with reduced sizes present some advantages, including (1) a large specific surface area, which translates into a greater number of active sites available for MET adsorption and photocatalytic reactions at the catalyst–water interface. (2) A short diffusion distance for charge carriers: in particles with small dimensions, photogenerated electrons (e^−^) and holes (h^+^) have a short distance to travel to reach the surface and participate in redox reactions, which can decrease the probability of e^−^/h^+^ recombination, a limiting factor in photocatalysis. (3) Quantum size effects: for semiconductor particles with particle sizes below a certain threshold (typically <10–20 nm), quantum effects can be observed, such as an increase in the band gap and a higher redox potential of the charge carriers. An average particle size of 11.68 nm is at the limit or within the range where these effects could begin to become significant, potentially increasing the oxidative power of the catalyst.

**Figure 3:**
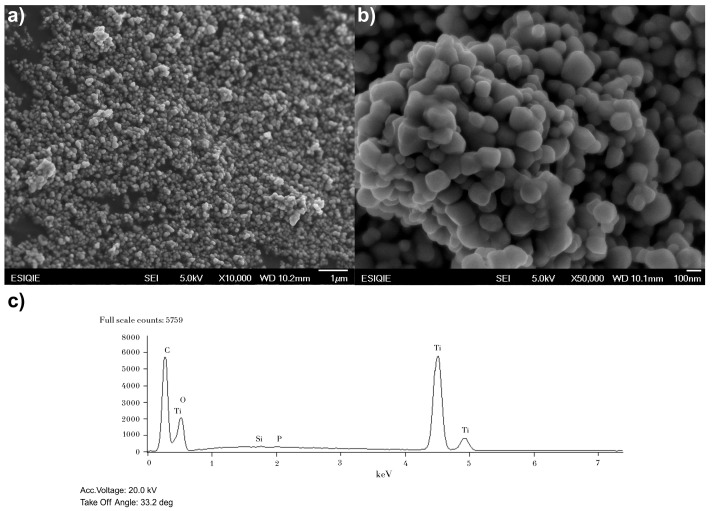
Characterization of Fermont catalyst: (**a**) SEM image with a 1 µm scale, (**b**) SEM image with a 100 nm scale, and (**c**) EDS analysis results.

The SEM analysis for the Fermont catalyst (Figure 3) showed that its particles agglomerate into large, dense structures with a crystal size of 80.71 nm. This size is significantly larger than that for P25, which reduces the available active surface and increases the probability of recombination. Although its band gap is the lowest at 3.23 eV, the absence of rutile and the unfavorable morphology drastically limited its efficiency.

SEM micrographs (Figure 3a,b) show particles forming spherical or globular aggregates of larger sizes that are composed of smaller, compacted primary particles. The morphology is different from that of P25, appearing denser and with more defined agglomerates.

**Figure 4:**
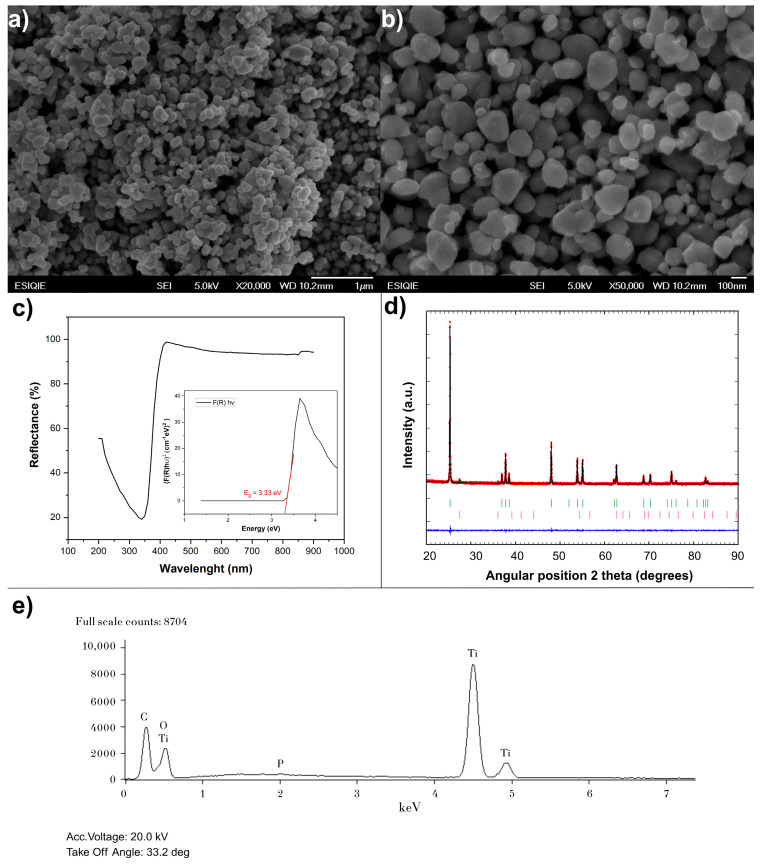
Characterization of Sigma-Aldrich catalyst: (**a**) SEM image with a 1 µm scale, (**b**) SEM image with a 100 nm scale, (**c**) band gap calculation graphs, (**d**) XRD pattern, and (**e**) EDS analysis results.

The XRD pattern for the Sigma-Aldrich catalyst is shown in Figure 4. XRD peaks correspond to those reported in the past for the anatase and rutile phases [24]. The P25 catalyst was previously reported to present peaks in angles characteristic for anatase and rutile polymorphs [24], while the Fermont TiO_2_ presented only peaks in angles corresponding to anatase [25]. The SEM micrographs for the TiO_2_ from Sigma-Aldrich (Figure 4a,b) show agglomerated particles, with a morphology that appears intermediate between the fine dispersion (at the primary level) of P25 and the larger aggregates of Fermont.

The calculated band gap for the Sigma-Aldrich catalyst was 3.33 eV (Figure 4c), which means the semiconductor needs radiation with a wavelength lower than 372 nm to achieve electron migration from the valence to conduction band. Since the amount of UV radiation that reaches the Earth is greatly affected by the atmosphere [26,27], it was not expected to yield the best results in solar radiation experiments. Nevertheless, the high percentage of anatase in the molecule must be considered (Table 1), as anatase presents an indirect band gap that is opposite to rutile, which has a direct band gap [28]. Considering this, differences are expected when working with the three catalysts, given their differences in anatase/rutile proportion.

The results obtained in this research demonstrate remarkable differences in photocatalytic activity between the catalysts, which can be attributed to the chemical, physical, and electronic properties of their anatase and rutile phases [29]. According to Zeng [30], the proportion of anatase and rutile polymorphs in P25 is 80/20. The difference with the results found here could be due to the different conditions in which the analysis was performed. 

According to the literature, anatase has a band gap of 3.2 eV, while rutile band gap is 3.0 eV [31,32]. Therefore, a difference in photocatalytic efficiencies was expected, as the anatase/rutile proportion in each catalyst differs. Semiconductor materials absorb photons with equal or greater energy than their band gap [33]. Thus, the band gap represents the energy needed to promote an electron from the valence to conduction band [34,35].

As well as the phase composition of TiO_2_ determining its photocatalysis degradation efficiency, the size and shape of the catalyst particle is also a determining factor [36]. It is known that the reactive sites on the surface of the catalyst are those that interact with the medium and the contaminant; this is where the redox reactions occur that facilitate the degradation of the contaminant. The larger the TiO_2_ surface area, the greater the number of reactive sites where pollutants can be adsorbed and react [36,37].

The atomic percentage reported in Figure 2c, Figure 3c and Figure 4e does not represent the true stoichiometry of the TiO_2_ compound but rather indicates the elemental composition of each catalyst. The discrepancy between the theoretical atomic composition (33 at% Ti and 66 at% O) and the experimental data (approximately 20 at% Ti and 80 at% O) is a common deviation in EDS analyses and does not signify an error in the sample composition. Instead, it is attributed to the inherent limitations of the technique, such as (1) difficulty in quantifying light elements: elements with a low atomic number, such as oxygen, are challenging to quantify with precision using EDS. The peak overlap in the spectra also affects accuracy. (2) Presence of impurities: although minimal, the presence of other elements like silicon (Si) and phosphorus (P) was detected in the EDS analyses, which affects the overall quantification. (3) Contamination: the absorption of surface moisture by the catalyst during sample preparation can inflate the reported oxygen percentage. In conclusion, the observed stoichiometric deviation does not reflect an incorrect catalyst composition but rather demonstrates the inherent limitations of the EDS analysis technique [38]. Nevertheless, the results are entirely valid for the purpose of this study, which is to confirm the elemental composition of the catalysts.

Regarding the EDS analysis, the corresponding data for the P25, Fermont, and Sigma-Aldrich catalysts are presented in Figure 2c, Figure 3c and Figure 4e, respectively. These analyses show the elemental composition of each catalyst. In P25, silicon (Si) was detected at 0.27% by weight. In Fermont, traces of silicon (Si) and phosphorus (P) were found at 0.10% and 0.13% by weight, respectively. In Sigma-Aldrich, phosphorus (P) was also detected at 0.14% by weight. The presence of these elements is due to them being byproducts of the manufacturing process or residual impurities. Although TiO_2_ is the main component, the industrial production of commercial catalysts does not always guarantee 100% purity. The presence of these elements in such low percentages does not have a significant impact on the photocatalytic activity of the TiO_2_ [39,40].

The performance of nano-photocatalysts is highly affected by particle size [24,41,42], as it affects the optical properties of the semiconductors [43]. The high degradation efficiency when using P25 could be due to the high rutile proportion, which has a lower band gap than anatase, combined with the smaller crystal size of the semiconductor. Nevertheless, the absorption band shifts towards a longer wavelength region as the crystal size in the samples increases, meaning a lower band gap energy [36]. This affirmation coincides with results presented here, where the catalyst with the largest crystal size, Fermont TiO_2_, has the lowest band gap.

In summary, comparing the three catalysts demonstrates that photocatalytic performance does not depend on a single property but rather on the optimal combination of phase composition (anatase/rutile), particle size, and morphology [36,42].

### 3.2. Photolysis Experiments

Control photolysis experiments performed in the dark did not show a remarkable COD decrease—the highest being 15% under acidic pH (Figure S3). Such results can be attributed to adsorption of the pollutant over the glass surface of the catalyst. Predicting the sorption behavior of organic cations is particularly challenging, as cation exchange is influenced by a wide range of factors, including the properties of the aqueous phase, the sorbent, and the sorbate [44].

The results from the UV-Vis absorption spectra confirm absorption peaks at 223.2 and 275.2 nm, meaning direct photolysis of the chemical is difficult as the solar spectrum reaching the Earth’s surface and the absorption spectra do not overlap. Most pharmaceutical compounds are photoactive because their structural compositions consist of aromatic rings and other functional groups that can either absorb solar radiation or react with photogenerated byproduct species in natural water [45].

**Figure 5:**
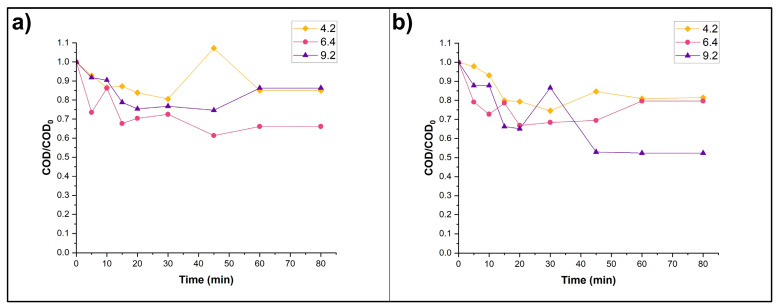
Photolytic degradation of MET under three different pH conditions: (**a**) without H_2_O_2_ addition and (**b**) adding 4 mM of H_2_O_2_ into the initial experimental volume.

When investigating photocatalysis, it is essential to differentiate the effects of photolysis, as this is anticipated to address the degradation of substances that is primarily driven by the catalyst’s action [46]. Photolysis experiments, performed in the FPR but in the absence of catalyst, showed the highest COD removal of 50% in pH 9.2 when H_2_O_2_ was added to the experiment (Figure 5).

The half-life of MET when exposed to sunlight has been reported to be several hundred hours [47]. Photolysis is a slow option for degrading the contaminant; additionally, its natural degradation in the environment is insufficient. In the past, it has been reported that MET does not undergo photolysis under solar radiation [48]. Direct photolysis is possible when chromophoric groups can absorb light at the wavelengths present in sunlight (λ > 290 nm). However, this process is often inefficient for organic compounds due to the limited overlap between their absorption spectra and that of solar radiation. In surface waters, indirect photolysis occurs via light absorption by some photosensitizers, such as nitrate/nitrite (NO_3_^−^/NO_2_^−^), and/or chromophoric dissolved organic material [45]. The degradation of MET can occur in two ways [49]:

MET + hv → intermediates(11)

HO∙ + MET → intermediates → CO_2_ + H_2_O (12)

Photolysis of MET has previously been reported when working with UVC lamps [50], which, as stated before, overlap with MET absorption spectrum and cause its breakage. Nevertheless, as this study was conducted under solar radiation, the path portrayed in Equation (11) is not possible, so photolysis must be due to the reaction from MET and hydroxyl radicals in the solution (Equation (12)).

### 3.3. Photocatalysis Experiments: Effects of Catalyst, pH, and H_2_O_2_

**Figure 6:**
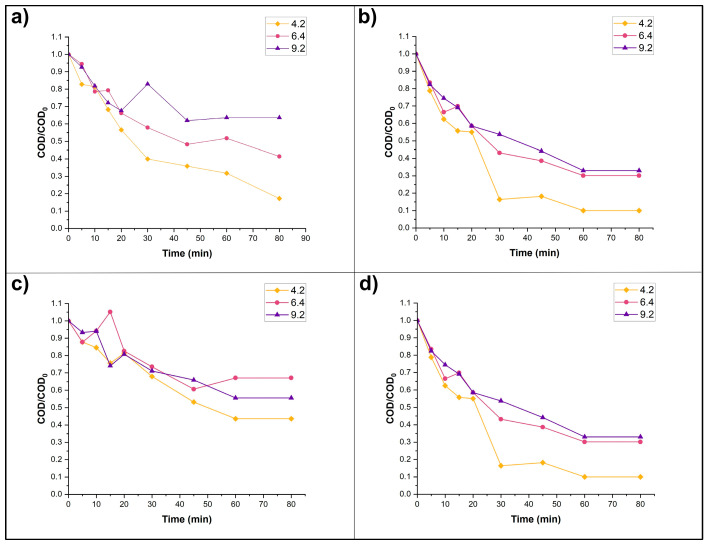
Photocatalytic MET degradation results with three different catalysts under three different pH conditions: (**a**) P25 without H_2_O_2_ addition, (**b**) P25 with H_2_O_2_ addition, (**c**) Sigma-Aldrich without H_2_O_2_ addition, and (**d**) Sigma-Aldrich with H_2_O_2_ addition.

Photocatalysis experiments were performed using the three catalysts in order to compare their efficiency under the same experimental conditions. Control photocatalysis experiments performed in the dark showed levels of removal ranging from 0 to 18% (Figure S3). The results for the P25 and Sigma-Aldrich catalysts are shown in Figure 6. The P25 catalyst, when H_2_O_2_ was added, exhibited better results in terms of COD removal (90%), as well as mineralization followed by TOC: a final TOC of 7.06 mg L^−1^ when using P25 catalyst, pH 4.2, and H_2_O_2_ addition, compared to a final TOC of 22.59 mg L^−1^ under pH 4.2 and with H_2_O_2_ addition when using Sigma-Aldrich catalyst.

MET is a weak acid with a pK_a_ value of around 9.7 [44,47]. Meaning, when the pH is under this value, its surface is positively charged. All three pH solutions tested were lower than the pK_a_ of the MET, so in all experiments, the pollutant was in a cationic state. Thus, the PZC of the catalyst governs the electrostatic interactions between the pollutant and the catalyst.

The addition of H_2_O_2_ provides a path for the formation of free HO· when reacting with the electron hole pair [31,51]. Nevertheless, the HO· reaction could be followed by the formation of OH^−^, which is not as reactive (Equations (13)–(15)):

H_2_O_2_ + e^−^ → HO∙ + HO^−^ (13)

HO· + e^−^ → HO^−^ (14)

H_2_O_2_ + 2e^−^ → 2HO^−^ (15)

It is well known that the addition of H_2_O_2_, by providing a path for HO· formation, improves photocatalysis [52]. Moreover, H_2_O_2_ is a better electron acceptor than O_2_ and avoids electron-hole recombination [53,54]. In all cases, H_2_O_2_ improved MET degradation (Figure 6), a result expected according to the literature.

A slight apparent increase in pollutant concentration was previously reported in photocatalytic degradation of MET [55,56] and Diclofenac [56]. This effect may be due to the breakdown of MET into shorter molecules with different levels of oxidation resistance. However, measurement or identification of byproducts was not an objective of the present study, so it is not possible to verify this as a response to the apparent increase in COD in this study.

When working with mixed-phase TiO_2_, it is important to consider some of the characteristics of each phase. Anatase, with a band gap of 3.2 eV [57], presents marked advantages: higher carrier energies (e^−^/h^+^), which favor stronger redox reactions such as the generation of HO∙; lower charge recombination compared to rutile, improving catalytic efficiency under UV; and high oxidative power, which is ideal for the degradation of recalcitrant contaminants. In terms of disadvantages, it is worth mentioning that it only absorbs UV light (λ < 387 nm), which limits its use in sunlight, as only around 5% of the solar spectrum is UV [23,58].

Rutile, in contrast, has a lower band gap (around 3.0 eV), so it absorbs radiation towards the visible region, λ < 413 nm [57], making better use of sunlight. Nevertheless, it has a greater tendency towards electron-hole recombination, reducing efficiency and lowering the redox potential of the carriers, limiting its ability to degrade certain contaminants.

### 3.4. Kinetic Analysis

It was determined using kinetics calculations the that degradation of MET in aqueous solution via solar photocatalysis follows a first-order reaction under acidic pH conditions (4.2), both without (0 mM H_2_O_2_) and with a concentration of 4 mM of H_2_O_2_, which is consistent with the reports by Yang et al. [59] and Romero et al. [46]. Photolytic degradation of most organic compounds follows pseudo-first-order kinetics [31]. The degradation of MET here presented was also adjusted to a first-order reaction.

Kinetic analysis was applied to the experiments in which a degradation percentage above 50% was obtained. The total organic carbon (TOC) test was performed only for the final measurement of the aforementioned experiments.

As can be seen in Table 2, the best reaction constant (k = 0.0486 min^−1^) was obtained in the experiment carried out with the P25 TiO_2_ catalyst at an acidic pH of 4.20 and with 4 mM H_2_O_2_. The highest percentage of COD removal was also obtained (90%) in these conditions. A k of 0.0283 min^−1^ was calculated for the experiment in which the Sigma-Aldrich TiO_2_ was used at an acidic pH (4.2) and 4 mM H_2_O_2_ was added. This data agrees with that reported by Malato et al. [60], who established that the highest k value corresponds to the highest percentage of degradation obtained. Thus, it is understood that the value of the rate constant will be lower in experiments where there are lower percentages of degradation. 

### 3.5. Statistical Analysis Results

Statistically speaking, there were no significant differences due to pH at any of the three levels (acid, base and neutral) at any sampling time. Changes in pH not leading to statistically significant results was not anticipated, as a pH of 9.2 was expected to yield remarkably better results when considering surface charges and charge interaction among molecules. When the pH of a solution is higher than the PZC of TiO_2_, its surface is negatively charged [61], which is what happens at experiments conducted at pH 9.2. At this pH, MET is cationic given its pK_a_, so the interaction between positively and negatively charged molecules should facilitate contact. Meanwhile, in experiments in pH 4.2 and 6.4 solutions, both substances are positively charged and so such interactions might be difficult, yielding less favorable results. Likewise, there were no significant differences in radiation at any sampling time, because all experiments were carried out under similar environmental conditions. On the other hand, for H_2_O_2_, there was only a significant difference at 5 min, where the mean was higher when 4 mM of H_2_O_2_ was added than in the absence of H_2_O_2_. The difference in means was significant for the three different TiO_2_ catalysts that were used for the degradation from minute 15 to the end of the experiment. The Fermont catalyst differs from the P25 catalyst, while the Sigma-Aldrich catalyst does not differ from either of the above. Likewise, the separation of the P25 catalyst from the other two catalysts is noticeable; this difference becomes evident after 15 min. From this period onwards, the P25 curve begins to drop until it reaches its lowest value at 80 min. The results obtained from this test agree with the percentages of MET degradation obtained (Table 2), as well as with those documented by Malato et al. [60], since, as previously mentioned, the P25 TiO_2_ photocatalyst has superior performance in the degradation of contaminants due to its crystalline structure that is approximately 80% anatase and 20% rutile, followed by the Sigma-Aldrich catalyst and then the Fermont catalyst. The response surface graphs, presented in Figure S4, allowed the authors to better understand the effect of pH and H_2_O_2_ addition on COD removal.

A possible explanation for such an effect is the higher band gap for anatase than for rutile, as P25 presents a higher proportion of rutile, while the Fermont TiO_2_ is formed only by anatase. It has been reported that, in mixed-phase catalysts, electron-hole recombination might be suppressed due to the transference of photogenerated charges between different TiO_2_ polymorphs [62,63]. Moreover, a smaller crystal size important to the success of the P25 catalyst.

The nature of the band gap (direct or indirect) in the TiO_2_ phases (anatase and rutile) plays a crucial role in their photocatalytic efficiency, as it affects light absorption and charge carrier recombination (e^−^/h^+^). In semiconductors with an indirect band gap (anatase), the transition of an electron from the valence band (VB) to the conduction band (CB) requires a change in crystalline momentum besides energy, meaning carrier lifetimes (e^−^/h^+^) are longer, since recombination is less likely as it requires a phonon to conserve momentum [64]. This explains why anatase typically shows greater photocatalytic activity than rutile under UV radiation, despite its lower light absorption.

## 4. Conclusions

Three TiO_2_ catalysts were tested to determine if they yielded different results when degrading MET in photocatalytic experiments. According to the results, the type of catalyst used has a statistically significant effect on COD removal after 15 min of experimentation. The highest levels of degradation were reached at 60 min when experiments were carried out at a pH of 4.2 and with the addition of 4 mM H_2_O_2_, where a COD decrease of 90% was reached when using P25 as a photocatalyst (k = 0.0486 min^−1^, t_1/2_ = 14.3 min, and final TOC of 7.06 mg L^−1^). P25 is a well-known catalyst that exhibits promising degradation result for a variety of different organic pollutants.

The pH of the initial solution did not have a statistically significant effect, even though one was expected given the charge interactions between the surface of the catalyst and the pollutant. H_2_O_2_, a well-known oxidant that was expected to avoid electron-hole recombination, was demonstrated to be effective in increasing pollutant degradation, resulting in statistically significantly shorter reaction times.

The higher proportion of rutile and the smaller crystal size in the P25 catalyst are important factors for the success of this semiconductor as a catalyst. Anatase (indirect band gap) has lower photon absorption but greater charge separation, which favors prolonged redox reactions. Rutile (direct band gap) absorbs more photons but undergoes greater recombination, limiting its catalytic efficiency.

MET, a widely used drug for the treatment of different diseases, can be effectively degraded by photocatalysis, yielding favorable mineralization results in 80 min of reaction time. However, the formation of byproducts due to this degradation deserves further study, as does the application of this technology on a larger scale.

## Figures and Tables

**Table 1:** Crystallographic properties of TiO_2_ P25, TiO_2_ Sigma-Aldrich, and TiO_2_ Fermont from XRD.

	Phase	Spatial Group **	Phase%	ICCD Card	Cristal Size (nm)	Band Gap (eV)
TiO_2_ P25 *	Anatase	I 41/a m d [141]	85.27	00-021-1272	20.97	3.3
	Rutile	P42/m n m [136]	14.73	01-070-7347	33.96	
TiO_2_ Sigma-Aldrich	Anatase	I 41/a m d [141]	96.81	00-021-1272	58.6	3.33
	Rutile	P42/m n m [136]	3.19	01-070-7347		
TiO_2_ Fermont *	Anatase	I 41/a m d [141]	100	00-021-1272	80.71	3.23

* Data for the P25 and Fermont catalysts was taken from González-Burciaga et al. [24]. ** Spatial group numbers shown in brackets.

**Table 2:** Kinetic data of photocatalytic experiments performed under pH 4.2 with P25, Fermont, and Sigma-Aldrich photocatalysts.

Catalyst	H_2_O_2_ Addition (mMol/L)	K (min^−1^)	Half-Life Time (min)	COD Removal (%)
P25	0	0.0249	27.8	83.0
	4	0.0486	14.3	90.0
Sigma-Aldrich	0	0.0185	37.5	56.4
	4	0.0283	24.5	63.0
Fermont	0	*	*	16.0
	4	*	*	21.1

* Kinetic parameters for MET degradation using the Fermont catalyst are not presented as degradation was remarkably lower than that for the other catalysts.

## Data Availability

The original contributions presented in this study are included in the article/Supplementary Materials. Further inquiries can be directed to the corresponding authors.

## Supplementary Materials

The following supporting information can be downloaded at: https://www.mdpi.com/article/10.3390/nano15181445/s1, Figure S1: Metoprolol molecule representation (C_15_H_25_NO_3_). Red atoms correspond to C, green atoms correspond to N, and blue atoms correspond to O.; Table S1: Elution gradient used in UPLC analysis; Figure S2: Solar radiation registered in Durango City, Mexico, in 2024, the year when the experiments were carried out; Figure S3: Results from control experiments performed in the dark, with acidic pH conditions and the addition of 4 mM of H_2_O_2_; Figure S4: Response surface graphs for the three tested catalysts.

## Abbreviations

The following abbreviations are used in this manuscript:

MET	Metoprolol
WWTP	Wastewater treatment plant
AOP	Advanced oxidation process
XRD	X-ray diffraction
NIR	Near infrared
SEM	Scanning electron microscopy
TEM	Transmission electron microscopy
DAD	Diode array detector
FPR	Flat-plate reactor
COD	Chemical oxygen demand
PZC	Point of zero charge
EDS	Energy dispersive spectroscopy
TOC	Total organic carbon
