# Supplementary material for: Comparative Efficiencies of TiO2 Photocatalysts on β-Blocker Metoprolol Degradation by Solar Heterogeneous Photocatalysis
Source: Nanomaterials (Basel). 2025 Sep 19;15(18):1445. doi: 10.3390/nano15181445 (PMC12472614; doi:10.3390/nano15181445)
Supplement: Supplementary file 1 [file nanomaterials-15-01445-s001.zip › nanomaterials-3842800-SI.pdf]

Supplementary material for:

Comparative efficiencies of TiO_2 photocatalysts on β-blocker metoprolol degradation by solar heterogeneous photocatalysis.

Irma C. Torrecillas-Rodríguez [1], Francisco Rodríguez-González [2], Daniel Tapia-Maruri [2], Héctor J. Dorantes-Rosales [3], José L. Molina-González [4], Cynthia M. Núñez-Núñez [5,*] and José B. Proal-Nájera [1,*].

1 Instituto Politécnico Nacional. Centro Interdisciplinario de Investigación para el Desarrollo Integral Regional Unidad Durango. Durango, Dgo., C.P. 34220, México; itorrecillasr1800@alumno.ipn.mx (I.C.T.-R.), jproal@ipn.mx (J.B.P.-N.)
2 Instituto Politécnico Nacional. Centro de Desarrollo de Productos Bióticos. Yautepec, Mor., C.P. 62731, México; frrodriguezg@ipn.mx (F.R.-G.), dmaruri@ipn.mx (D.T.-M.)
3 Instituto Politécnico Nacional. Escuela Superior de Ingeniería Química e Industrias Extractivas. Ciudad de México., C.P. 07300, México; hdorantes@ipn.mx
4 Universidad de Salamanca. Centro de Investigación y Desarrollo Tecnológico del Agua. Salamanca., 37080, España; jlmolina@usal.es (J.L.M.-G.)
5 Universidad Politécnica de Durango. Ingeniería en Tecnología Ambiental. Durango, Dgo., C.P. 34300, México.
* Correspondence: cynthia.nunez@unipolidgo.edu.mx; Tel.: (C.M.N.-N.), jproal@ipn.mx; Tel. : (J.B.P.-N.).

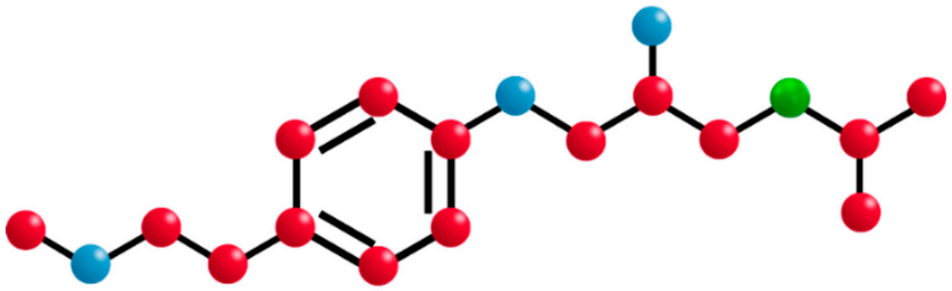

Figure S1. Metoprolol molecule representation (C15H25NO3). Red atoms correspond to C, green atoms correspond to N and blue atoms correspond to O.

Table S1. Elution gradient used in UPLC analysis.

Time (min)	Flux (mL/min)	% A	% B
0	0.3	95	5
2	0.3	95	5
2.01	0.5	80	20
2.4	0.5	80	20
2.5	0.6	75	25
4	0.4	72	28
5	0.5	72	28
7	0.5	50	50
8	0.5	25	75
8.05	0.5	10	90
10	0.5	10	90

A: 0.05% trifluoroacetic acid

B: acetonitrile

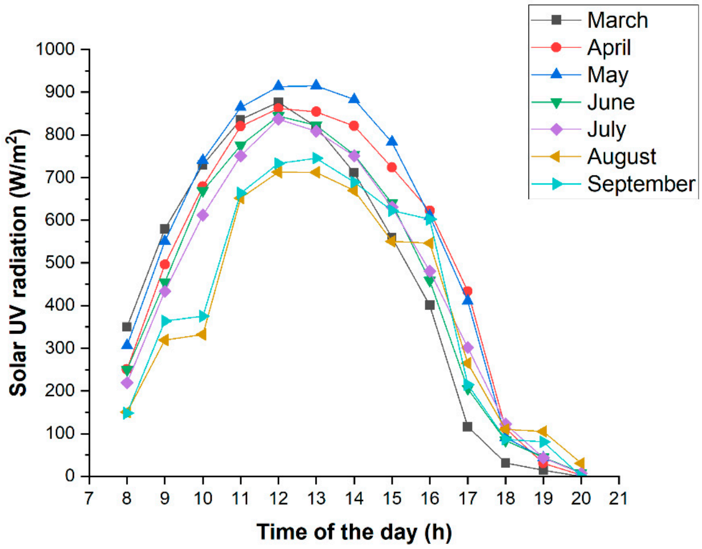

Figure S2. Solar radiation registered in Durango City, Mexico in 2024, the year where the experimentation was carried out.

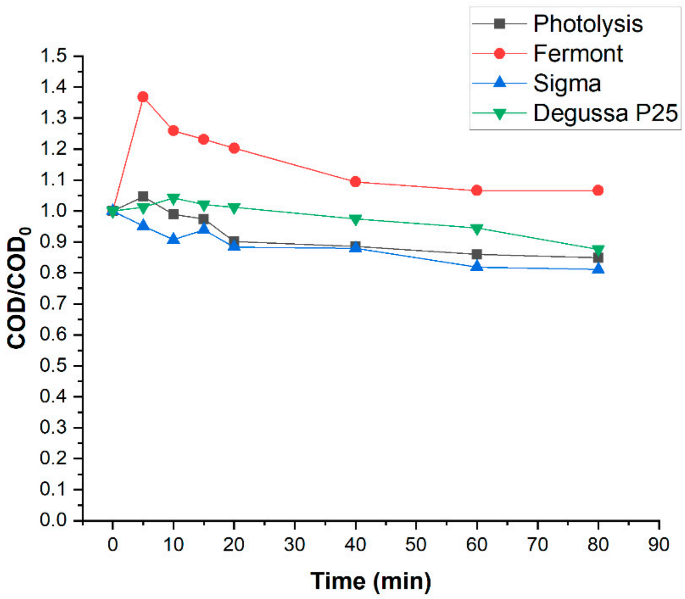

Figure S3. Results from control experiments performed under dark conditions and acidic pH with addition of 4 mM H_2O_2

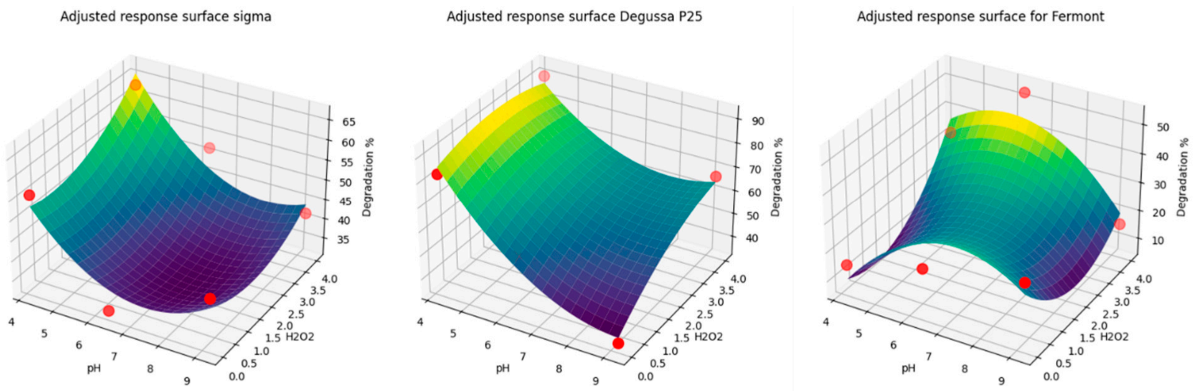

Figure S4. Response surface graphs for the three tested catalysts
